# Enhancing Health Through Access to Nature: How Effective are Interventions in Woodlands in Deprived Urban Communities? A Quasi-experimental Study in Scotland, UK

**DOI:** 10.3390/su11123317

**Published:** 2019-06-15

**Authors:** Catharine Ward Thompson, Aldo Elizalde, Steven Cummins, Alastair H. Leyland, Willings Botha, Andrew Briggs, Sara Tilley, Eva Silveirinha de Oliveira, Jenny Roe, Peter Aspinall, Richard Mitchell

**Affiliations:** 1 OPENspace, University of Edinburgh, Edinburgh EH3 9DF, UK; 2 Adam Smith Business School, University of Glasgow, Glasgow G12 8QQ, UK; 3 Department of Public Health, Environments & Society, London School of Hygiene & Tropical Medicine, London WC1H 9SH, UK; 4 Medical Research Council/Chief Scientist Office (MRC/CSO) Social and Public Health Sciences Unit, University of Glasgow, Glasgow G2 3AX, UK; 5 RTI Health Solutions, 3040 East Cornwallis Road, P.O. Box 12194, Research Triangle Park, Durham, NC 27709, USA; 6 Health Economics and Health Technology Assessment, Institute of Health and Wellbeing, University of Glasgow, Glasgow G12 8RZ, UK; 7 Center for Design and Health, University of Virginia, Charlottesville, VA 22903, USA

**Keywords:** Natural environment, mental health, stress, deprived urban communities, quasi-experimental, natural environment interventions, forestry, woodlands, community forest management

## Abstract

High prevalence of poor mental health is a major public health problem. Natural environments may contribute to mitigating stress and enhancing health. However, there is little evidence on whether community-level interventions intended to increase exposure to natural environments can improve mental health and related behaviours. In the first study of its kind, we evaluated whether the implementation of a programme designed to improve the quality of, and access to, local woodlands in deprived communities in Scotland, UK, was associated with lower perceived stress or other health-related outcomes, using a controlled, repeat cross-sectional design with a nested prospective cohort. Interventions included physical changes to the woodlands and community engagement activities within the woodlands, with data collected at baseline (2013) and post-intervention (2014 and 2015). The interventions were, unexpectedly, associated with increased perceived stress compared to control sites. However, we observed significantly greater increases in stress for those living >500 m from intervention sites. Visits to nearby nature (woods and other green space) increased overall, and moderate physical activity levels also increased. In the intervention communities, those who visited natural environments showed smaller increases in stress than those who did not; there was also some evidence of increased nature connectedness and social cohesion. The intervention costs were modest but there were no significant changes in quality of life on which to base cost-effectiveness. Findings suggest factors not captured in the study may have contributed to the perceived stress patterns found. Wider community engagement and longer post-intervention follow-up may be needed to achieve significant health benefits from woodland interventions such as those described here. The study points to the challenges in evidencing the effectiveness of green space and forestry interventions to enhance health in urban environments, but also to potential benefits from more integrated approaches across health and landscape planning and management practice.

## 1 Introduction

Living in urban environments, especially for those living in relative poverty, has been associated with high levels of stress and poor mental health [1,2]. Such non-communicable diseases form a growing part of the burden of ill-health in an increasingly urbanised society, and offer challenges for healthcare provision. Socio-ecological models of health acknowledge the potential for the physical environment to have pathogenic or salutogenic effects [3] but addressing today’s health challenges requires an integration of health issues into spatial and urban landscape planning, taking into account the potential for multiple health and environmental effects, and acknowledging possible unintended outcomes. There are many difficulties in determining how to guide and regulate such planning approaches in the context of other demands for sustainable and cost-effective urban development [4–6]. Prüss-Ustün et al. (2016, p. 474) have suggested that “Investing in environmental interventions pays off for governments; it reduces the transfer of hidden costs from other sectors to the health sector” [6]. However, little is known on whether environmental interventions can enhance mental health and, if so, what characterizes the planning and implementation of effective interventions. This paper addresses this challenge by assessing the effectiveness of environmental interventions in the area of urban green space, forestry and health, using a quasi-experimental approach to provide evidence that can inform government policy-makers, land owners, stewardship communities, practitioners and non-government organisations engaged in supporting community health and wellbeing.

## 2 Background

Multiple studies suggest that access to green or natural environments may play an important role in mitigating stress levels and contributing to better mental health [7,8]. Several causal pathways have been hypothesised, including physical activity, psychophysiological responses and the benefits of mental restoration associated with natural environments [9–11]. Population-level observational studies have shown that positive associations between mental health and access to nature are stronger among those of low socio-economic status (SES) compared to those of higher SES [12,13]. The evidence for the positive effects of interventions to enhance access to natural environments is based largely on experimental studies where participants were randomly allocated to activities (e.g., gardening, walking) in green spaces [14,15]. However, evidence for the impact of interventions involving a physical change to the environment is less clear. Where epidemiological studies have considered change over time in access to green space, either via people moving home [16] or via authorities improving the quantity and/or quality of neighbourhood green space [17], the findings have been equivocal and, in the latter case, enhanced green space was not associated with benefits to physical activity or self-reported health. There is very little evidence on how best to design natural environment interventions that encourage the local community to visit green space and that result in improved health at a population level [1]. This paper addresses this evidence gap by assessing whether physical and social (i.e., community engagement) interventions designed to enhance access to green space are associated with improved mental health. The full project report on which this paper is based is available at the National Institute for Health Research (NIHR) journals library [18].

**Figure 1:**
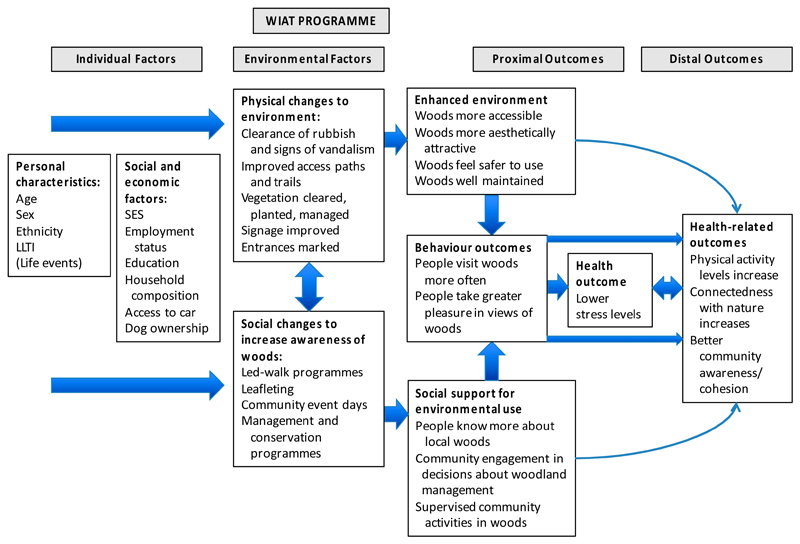
Logic model showing hypothesized pathways for the Woods In and Around Towns (WIAT) intervention programme. Note: This is a slightly modified version of a figure first published in Silveirinha de Oliveira et al., BMJ Open, 2013 [23].

Our study developed a logic model to consider how physical and social interventions focused on community natural environments might offer pathways to perceptions, behaviour, and different health outcomes, with a particular focus on reduced stress and mental health. Figure 1 shows how we conceptualized likely effects—both practical and psychological—of an enhanced physical environment and social activities designed to engage community members in visiting the natural environment. We considered that use of woods and views of them might directly contribute to reduced stress [8,19] but also that indirect pathways to reduced stress might include physical activity [20], psychological connection with nature [21], and enhanced community cohesion [22].

**Figure 2:**
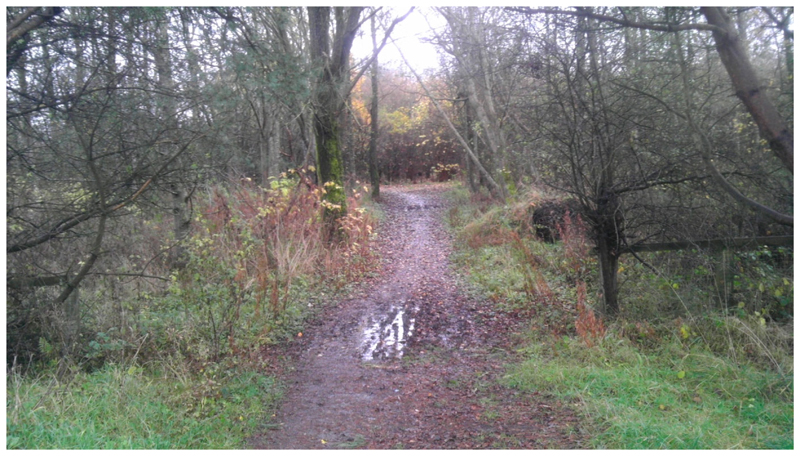
Typical woodland site pre-intervention. This shows this condition of a woodland site prior to the baseline survey being undertaken, with a poorly drained footpath and vegetation beginning to overhang the path. Reproduced with permission from OPENspace, University of Edinburgh.

**Figure 3:**
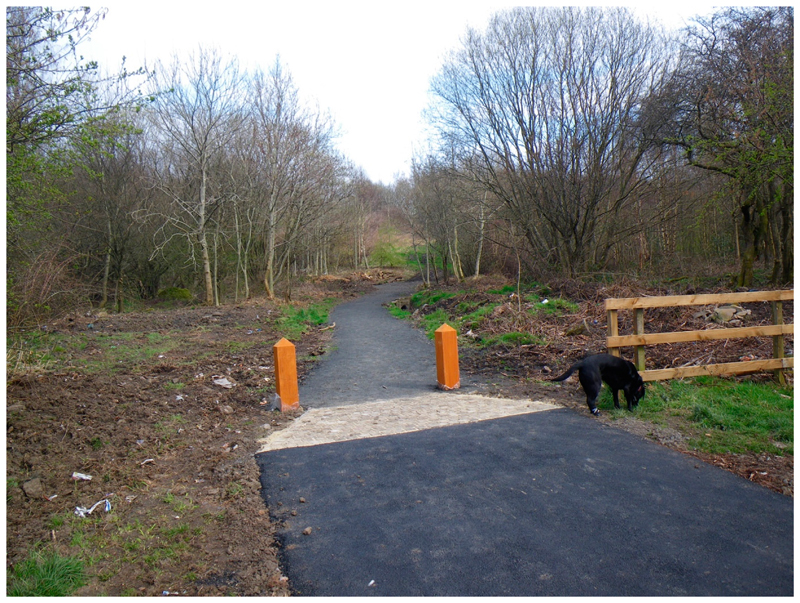
Typical woodland site post physical woodland intervention.

**Figure 4:**
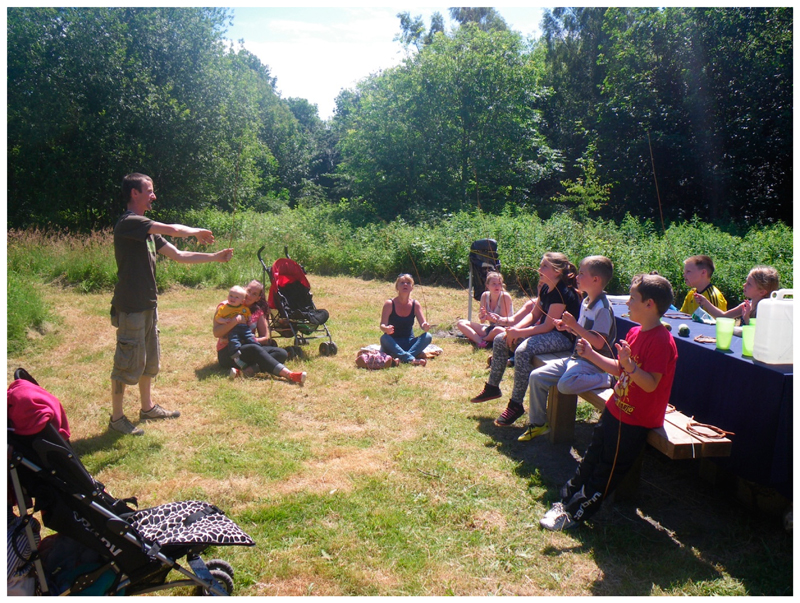
Typical social intervention to engage local communities in the woodlands.

This study took advantage of environmental interventions delivered by Forestry Commission Scotland (FCS) under their Woods In and Around Towns (WIAT) programme [24], to develop a quasi-experimental study. WIAT is targeted at woodlands near socially deprived urban communities; it aims to increase local residents’ contact with woodlands within 1km of settlements with a population of 2000 people or more, in order to enhance wellbeing and quality of life. The WIAT interventions involve two stages: Modest-scale physical changes to the woods to improve their attractiveness for use, consisting largely of footpath surfacing and drainage, improving entrances and clearing rubbish and overgrown vegetation; and community engagement events to attract the local community to use the woods, including ‘family fun days’, photography and environmental art workshops, and activities for school children. See Figures 2–4 for a description of the physical interventions and community engagement activities.

Physical interventions were designed to enhance sustainable management of the woodlands and improve onsite recreation facilities. They typically included new or resurfaced and drained footpaths, signage, and entrance features. They were undertaken over a period of eight months, between July 2013 and February 2014, to ensure completion at least two months before the Wave 2 community survey. Reproduced with permission from Forestry Commission Scotland.

Social interventions consisted of a programme of locally advertised and facilitated community-level activities and events, e.g., guided walks, ‘family fun’ days, ‘scavenger hunts’, and woodland based classes for schoolchildren, intended to promote the woodlands and increase their use. The social interventions started in June 2014 (four months after the physical interventions were completed), and took place over nine months, until March 2015. This timescale was somewhat compressed compared with typical WIAT interventions in order to ensure the interventions were completed at least two months before the final (Wave 3) community survey. Reproduced with permission from Forestry Commission Scotland.

## 3 Aims

We aimed to assess whether there were changes in mental health within deprived communities that received the interventions. First, we assessed whether the WIAT programme of interventions was associated with changes in perceived stress. Second, we assessed whether there were differential changes by gender and distance from the interventions. We also explored the impact of WIAT on people’s engagement with the woods and other natural environments; the effect of physical interventions alone versus both physical and social interventions; and other health-related outcomes, including health-related qualily of life, physical activity levels, connectedness to nature and community cohesion. The cost of the interventions in relation to the health-related consequences was also assessed.

## 4 Methods

### 4.1 Study Design

A pilot study with two communities that met the WIAT criteria in 2006–9 [25] indicated that the recruitment and retention of a longitudinal cohort of participants of sufficient power for the current study was unlikely to be successful. Most potential participants contacted in the pilot study were unwilling to make such a commitment. The design for the study described here therefore involved repeat, cross-sectional surveys of individuals resident in intervention and control communities, with three waves of data collection in the early summer of 2013 (baseline, Wave 1), 2014 (Wave 2) and 2015 (Wave 3) respectively, to assess outcomes. However, in order to maximise the value of data collected, we attempted to return to participants from the Wave 1 survey in Waves 2 and 3, unless Wave 1 respondents chose to opt out after receiving a post-survey thank-you letter which indicated how to do so. By this means, we obtained a smaller longitudinal cohort of participants who had responded in Wave 1 and in either or both of Wave 2 and 3 surveys; this cohort was nested within the cross-sectional surveys and was used for sensitivity analyses. Data on the nature and cost of undertaking environmental changes in woodlands and of community engagement activities were also collected for use in health economic analyses.

The study used mixed methods involving environmental audits by trained auditors and local community members, and focus groups post-intervention; these aided understanding of community perceptions of the interventions and any impact on behaviour and wellbeing.

### 4.2 Study Setting and Choice of Sites

We identified three pairs of sites (where each site comprised a woodland and its associated community) in central Scotland that were eligible for WIAT and were in areas within the worst 30% of deprivation in Scotland as measured by the Scottish Index of Multiple Deprivation (SIMD) [26]. The sites varied in size between a minimum of 4ha and a maximum of 24 ha. To reduce confounding, each pair of sites was matched as closely as possible by physical characteristics and by the social, economic, demographic and health characteristics of the population resident in a catchment area, defined as 1.5 km around each site [23]. Sites were assigned to the intervention arm of the study based on their readiness for management agreements between the FCS and landowners (predominantly local authorities), to ensure they would proceed and that no other interventions were planned. In collaboration with FCS, the timing of intervention delivery was pre-planned with physical environment interventions (Phase 1) undertaken over eight months in 2013–14 and community engagement interventions (Phase 2) undertaken over nine months in 2014–15 (see Figures 2–4). This allowed for surveys of residents to happen at the same time of year in each of the three waves of the survey, i.e., late April to July, and a minimum of two months post Phase 1 interventions in 2014 and post Phase 2 interventions in 2015. As an indication of the scale of the interventions, 650 m of path improvements were undertaken for the smallest site and 1900 m of path improvements for the largest. In total, across all three intervention sites, 62 community engagement activities were undertaken, with total numbers of participants per site varying between 120 and 434.

### 4.3 Outcome Measures

Our logic model (see Figure 1) guided the choice of outcome measures. The primary health outcome measure was stress, measured using the Perceived Stress Scale (PSS) [27].

(a) PSS comprises 10 items (e.g., feeling nervous and stressed; feeling on top of things; being angered because of things outside your control) measured on a 5-item response from ‘never’ to ‘very often’. The final score assesses perceived stress over the preceding month and can range from 0 (minimum level of stress) to 40 (maximum level of stress). There is no clinically recognised cut-off for PSS; it was chosen because it has been shown to be sensitive to differences in access to green space [8] and is sensitive to change over time in relation to therapeutic interventions [28].
  Secondary, self-reported outcome measures included the following:
(b) Health-related quality of life was measured using the self-report EuroQol measure (EQ-5D) [29] that captures five dimensions of health state: Mobility; self-care; usual activities; pain/discomfort and anxiety/depression. The original version of EQ-5D has three levels (3L) for each dimension with a minimum (best health) score of 1 and a maximum (worst health) score of 3 while the new version has five levels (5L) for each dimension with scores from 1–5 [30]. The original 3L version was used in Wave 1 and the new 5L version in Waves 2 and 3 (see Appendix A for further details).
(c) Physical activity (PA) was measured using the International Physical Activity Questionnaire short form (IPAQ-SF) [31], to record the duration (in minutes) and frequency (in days) for three activities: Walking; moderate-intensity; and vigorous-intensity PA. To obtain weekly minutes of walking, moderate and vigorous PA in terms of Metabolic Equivalent (MET) values, participants’ estimates of the average number of minutes of activity was calculated by multiplying the weekly frequency and the respective MET scores. To obtain a total PA score, the three activities were summed.
(d) Nature visits were measured by asking if participants were aware what their local woods (target of study) were like and had visited these woods or other local parks or green spaces in the last 12 months (yes/no) [32]. If they had visited local woods, they were asked for the average length of woodland visit (6 categories, from up to 15 minutes to >5hours); and frequency of these visits in summer (April-Sept) and winter (October-March) [33].
(e) Emotional connection to the natural world (connectedness with nature) was measured using the Inclusion of Nature in Self Scale, a graphic, 7-item scale showing increasingly overlapping circles representing ‘me’ and ‘nature’ [34].
(f) Social cohesion was measured based on three items from the English Citizenship Survey covering the trust of neighbours, neighbourhood belonging, and whether people in the neighbourhood pull together to improve things [35]. The final scores from each 4-point scale were amalgamated to produce a summary scale capturing participants’ community collective strength, ranging between 3 and 12.

A range of socio-demographic variables considered potential confounders for any intervention effect were also collected, as indicated in Table 1.

The study was approved by the University of Edinburgh, Edinburgh College of Art Research, Ethics and Knowledge Exchange Committee.

### 4.4 Recruitment

Individuals aged 16 or older, living within the intervention and control communities, within 1.5 km of the target woodland site, were eligible for the study. Participants for the core survey were selected from the Ordnance Survey’s Address Point dataset, which listed all addresses to which the postal service will deliver, distinguished between business and private addresses and provided the precise geolocation for each address. A random sample of eligible houses was drawn, stratified by straight-line distance from address to the WIAT-eligible woodlands (within 150 m, 300 m, 500 m, 750 m and 1500 m). Recruitment involved an introductory mailing sent to all potential core survey households, informing them of the forthcoming survey and giving recipients the opportunity to ask questions and/or to opt out of being contacted further. Interviews were undertaken with the first eligible adult to respond to the surveyor calling at a selected address. Face-to-face computer-assisted personal interviews (CAPI) were carried out in participants’ homes by a commercial survey agency.

The total sample of *n* = 6317 was achieved across three survey waves (Baseline *n* = 2117, Wave 2 *n* = 2098, and Wave 3 *n* = 2102), The overall response rate was 53% (Baseline = 50%, Wave 2 = 52%, Wave 3 = 59%). The level of cooperation, i.e. the proportion of successful interviews achieved once personal contact with a household had been made, was 70% (Baseline = 73%, Wave 2 = 74%, Wave 3 = 64%). After data cleaning using range, consistency and logic checks in order to confirm their quality, and identification and removal of abnormal patterns in some of the PSS scores (e.g., large numbers with a zero score, indicating no stress at all) associated with particular interviewers, the sample was reduced to *n* = 5460, used in subsequent analyses reported here. This sample contained a nested cohort (*n* = 609), used for sensitivity analysis, the size of which was determined by the extent to which repeat responses were obtained by the recruitment method described.

### 4.5 Analysis

We first investigated changes in primary and secondary outcomes through an Intention-to-Treat (ITT) approach to establish the *differential* impact on the intervention and control groups after physical interventions (survey Wave 2) and after both physical and community engagement interventions (survey Wave 3) compared to baseline, irrespective of the respondents’ interactions with green spaces. We considered the difference in differences by fitting an interaction term between intervention or control site and survey wave. This analysis explored whether a change from baseline in the outcome variables differed between intervention and control groups.

We then examined whether differences in change over time between the intervention and control groups also varied by gender and distance to the woods, as previous research has indicated gender differences in relationships between green space and health [8,36], as well as by distance to green and woodland space [37]. To further explore the relationship between the interventions and the primary outcome, we created a dichotomised variable based on whether or not participants had visited their local woods or other local parks or green space in the previous 12 months. Our analysis then asked whether any intervention effect differed according to this ‘nature visit’ variable. The overall effect of the intervention within each level of these three indicators (gender, distance to the woods, nature visits) was given by a joint test of interaction terms.

Because our sample included a proportion of individuals who participated in more than one wave, the analyses used a multilevel framework. This accounted for repeated observations nested within individuals, as well as spatial clustering. All models were adjusted for participants’ age, gender, life events, social class, education, working status, income coping, access to car, smoking status, disability, health status, dog ownership, children, and distance bands to local woods. Linear and logistic multi-level regression models were fitted appropriate to the analysed outcome.

There were missing data in 19% of sample responses. To reduce potential bias, due to missing data, we undertook multiple imputation to produce ten datasets using chained equations, with estimates combined using Rubin’s rules [38]. This formed the analytical dataset. The nested cohort sample characteristics broadly matched the full sample but had older people (aged 75+), more negative life events, lower education levels and more health limitations, and did not equally represent all sites. It provided the basis for a form of sensitivity analysis for findings from the cross-sectional data. All analyses were conducted using Stata version 14 (StataCorp LLC, College Station, TX, USA).

The economic evaluation drew on health outcomes from the core survey and on resource data collected directly from FCS. This costed the time commitment of members of the FCS team involved in supporting the physical and community engagement interventions, administration and monitoring of any contractors’ work, and the direct cost of delivering the interventions. Cost-consequences analysis (CCA) [39] was used to present the total cost of the interventions in relation to the primary and secondary outcomes of the interventions. An exploratory cost-utility analysis was also conducted from the EQ-5D responses for the WIAT interventions over the timescale of the study.

## 5 Site Audits and Qualitative Data

Changes in the nature and quality of each woodland site were monitored every six months throughout the study, using a site-based environmental audit tool developed by the research team and used both by ‘expert’ researchers and by volunteers recruited from each local community [32]. The audit tool consists of twenty-five items aggregated into seven domains: Neighbourhood quality; access/signage; woodland/green space quality; facilities; use; maintenance/management and security/safety. Each domain contains between two and six items; for example, the domain, ‘neighbourhood quality’ comprises the items *infrastructure, appearance, litter* and *maintenance* [25]. In addition to a simple, 5-point score for each domain of the audit tool, auditors could also add comments on any relevant aspect. These qualitative data were supplemented towards the end of the study by focus groups with local residents in the three intervention communities to gain additional insights into the perceptions, experience and impacts of the physical and social interventions. Audit scores were simply summed over the seven domains, while the qualitative data were analysed using both pre-determined and an inductive thematic analysis [40].

## 6 Results

### 6.1 Core Survey Characteristics

At baseline, the sample was 62% female and 38% male, with 58% in the two lowest occupational social class groups (classes IV and V, see Table 1); 57% of the sample were not in work and 21% were finding it difficult to cope on current income.

The paired intervention and control site populations were well matched at baseline on gender, age, working status and life events. Table 1 shows that the most significant differences at baseline were in relation to certain indicators of socio-economic status, i.e., educational qualification level and subjective income coping, where the intervention group reported poorer educational attainment and lower ability to cope on current income. This difference was also reflected in lower access to a car and (to a lesser extent) lower social class among the intervention group, which also had higher levels of smokers. The intervention group had fewer people living close to their local woodlands and more living at a distance of 750 m or more compared to the control group. This was a function of street layout and urban form, rather than of sample bias.

### 6.2 Primary Outcome: Stress Levels

Survey Waves 2 and 3 showed stress levels (PSS) in the intervention group had increased compared to the control group. In the unadjusted analyses, the magnitude of the intervention effect on PSS at Wave 2 was 1.77 (95% CI 0.93 to 2.61) and at Wave 3 was 3.91 (95% CI 3.07 to 4.75). After adjusting for the potential confounders, the intervention was associated with a difference in PSS at Wave 2 of 1.52 points (95% CI 0.78 to 2.27) and at Wave 3 of 3.58 (95% CI 2.85 to 4.31).

### 6.3 Differences in Primary Outcome by Gender and by Distance from Woods

Interaction terms were formally tested via a Wald Test, allowing assessment of whether coefficients of interactions were significantly different from zero. We then conducted a joint test to compute the overall effect within each category for distance bands and gender. Women had higher levels of stress than men (results not shown) and no significant gender differences were found for intervention effects on stress levels. A significant effect for the interventions on stress levels was associated with the distance of participants’ home from the local woods. Compared to the control group, intervention participants within the lower distance bands had a smaller increase in their stress levels (PSS) relative to those within the upper distance bands, at each survey wave. The difference between PSS estimates for the lower and upper distance bands widened between Waves 2 and 3, with a difference in PSS score from baseline of 2.43 for the lowest distance (up to 150 m) (95% CI 0.32 to 4.53) compared to 9.71 for the highest distance band (750–1500 m) (95% CI 7.28 to 12.13) by Wave 3.

### 6.4 Secondary Health and Wellbeing Related Outcomes

Table 2 shows the results with regard to secondary health and wellbeing outcomes.

Fully adjusted analysis of the intervention effect on health-related quality of life measured by EQ-5D showed a small, non-significant positive effect at Wave 2 and a small, non-significant negative effect at Wave 3.

**Figure 5:**
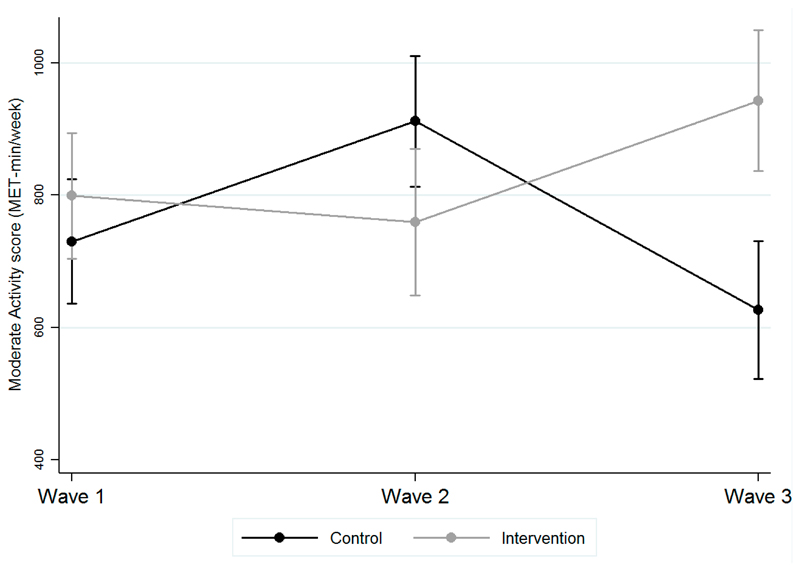
Predicted means of moderate intensity physical activity score comparing intervention and control groups over time. Note: Y-axis shows moderate intensity physical activity score in MET-min/week, and the x-axis the wave of the survey. An increase in the MET score denotes higher intensity physical activity. Controlled for different individual-level characteristics.

Compared with the control group, the intervention group was associated with a significant decrease in moderate PA at Wave 2, but a significant increase in moderate PA by Wave 3 (see Table 2). Figure 5 illustrates the interventions were associated with a change in moderate intensity PA of 144.7 METs from baseline (799.2, 95% CI 704.8 to 893.7) by Wave 3 (943.9, 95% CI 837.3 to 1050.5), compared with a control group change of –104.6 METs (baseline: 726.3, 95% CI 632 to 820.1; Wave 3: 621.7, 95% CI 517.6 to 725.9).

Interventions were associated with significantly greater awareness of the target local woods (OR 3.1, 95% CI 2.15 to 4.46 at Wave 3) but not with any significant change in length or frequency of visits to these woods. However, interventions were associated with significant differences in visits to nearby nature more generally, i.e., to green spaces, including the local woods. The intervention group showed significantly more people making nature visits at least once/year at Wave 3, compared with the control group (OR 2.69, 95% CI 1.9 to 3.81).

Table 2 shows that, after a significant decrease in connectedness to nature at Wave 2, the interventions were associated with a highly significant increase in connectedness to nature by Wave 3 compared to the control group. The interventions were also associated with a highly significant increase in social cohesion at Wave 2 and Wave 3 in comparison with the control group (see Table 2).

### 6.5 Using the Logic Model to Understand Outcomes

To explore whether the interventions’ association with increased nature visits moderated changes in stress, we introduced the nature visits variable into the model. The magnitude of the intervention effect on PSS for the intervention group who did *not* visit natural environments was 3.04 points at Wave 2 (95% CI 2.00 to 4.07) and 4.97 at Wave 3 (95% CI 3.95 to 5.99). By contrast, the magnitude of effect for intervention site participants who *did* visit natural environments showed no significant change at Wave 2 (PSS –0.06, 95% CI –1.12 to 0.99) and a smaller PSS increase of 1.94 at Wave 3 (95% CI 0.89 to 3.00). We found no significant changes in PA conditioned on nature visits.

These analyses suggest that visits to natural environments do not explain the association between the intervention and increases in perceived stress, which appears to be the result ot other influences. Rather, nature visits may moderate the effect of stressors. However, the analyses suggest that visits to natural environments do not moderate the association between the intervention and increases in moderate PA; it is thus unclear what pathway contributed to these changes in PA levels.

### 6.6 Economic Evaluation

The cost of the WIAT interventions is summarised in Table 3, split by external payments made by FCS and the internal staff time taken to manage the interventions. The cost per individual was calculated based on the eligible population (*n* = 20,472) of the intervention communities. This resulted in the average cost per person of £7.68, (95% CI £7.67–£7.69) for the physical intervention in Wave 2 and £11.80, (95% CI £11.79–£11.82) for both physical and social interventions in Wave 3.

The cost-consequences analysis compared these costs to the primary and secondary outcomes reported above and in Table 2 without explicit weighting of those outcomes. Interpretation is constrained by the fact that the only significant beneficial effects found for the interventions were for the secondary outcomes of moderate PA, connectedness to nature and social cohesion, while the primary outcome of PSS was negative. However, an exploratory cost-utility analysis (CUA) that included an assessment of uncertainty over the timeframe of the WIAT programme was conducted. The CUA compared the incremental expected cost of the physical intervention and both the physical and social interventions per individual in the eligible population with estimated quality-adjusted life years (QALYs) gained from the intervention, based on the adjusted difference in differences analysis of the health-related quality of life (HRQoL) utilities of the EQ-5D from Table 2. To account for the impact of time on costs and HRQoL utilities that happened at different times, a discounting rate of 3.5% was used as per National Institute for Health and Care Excellence (NICE) guidelines [41]. However, this discounting was inconsequential because the time horizon for economic evaluation was so short. The QALYs were calculated using the area under the curve approach which assumes linear interpolation in the change in HRQoL utilities between the waves [42,43]. Details of QALY calculations can be found in the full study report [18].

The CUA suggested that at Wave 2 the cost per QALY was £935, (95% CI £399 per QALY to dominated, i.e., dominated by higher cost and fewer QALYs than the control) for physical interventions while at Wave 3, the cost per QALY was £662, (95% CI £206 per QALY to dominated) for both social and physical interventions.

### 6.7 Sensitivity Analyses

In addition to the analysis on the basis of imputed data, complete case analyses were also conducted; they confirmed the findings reported above. As a further test of sensitivity, the cohort sample was analysed using the same approach as for the full, cross-sectional sample (see Appendix B). The findings were confirmed for PSS outcomes, PA and awareness of the local woods reported above. There were no significant intervention effects found for connectedness to nature or social cohesion, unlike in the full sample.

### 6.8 Site Audits and Qualitative Data

The environmental audit scores showed that the interventions were perceived as significantly enhancing the quality of the woodlands (*p* < 0.001), by both expert and community auditors, and this was true regardless of seasonality. After both phases of intervention were completed, the intervention sites were considered of significantly higher quality than the control sites. However, for the community auditors (but not the expert auditors), this had also been true at baseline.

The qualitative data indicate that the interventions were highly appreciated by community members, although these were often participants who already visited the woods regularly. Positive responses to the intervention included walking, appreciation of wildlife and nature (especially for children), and enjoyment of peace and quiet. There was also evidence of positive social engagement and community benefits. Negative comments largely focused on vandalism, litter and dog faeces, overgrown vegetation and deterioration of footpaths that reflected a lack of maintenance after the interventions were completed.

## 7 Discussion

**Figure 6:**
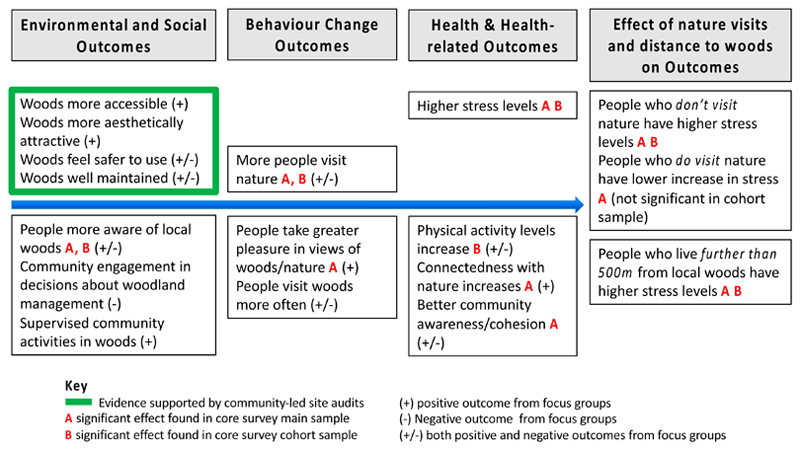
Summary of study post-intervention outcomes, i.e., after physical changes to the environment and social changes to increase engagement with local woods.

Figure 6 summarises the key findings of the study. Neither the physical interventions alone, nor the addition of social interventions under the WIAT programme, were sufficient to achieve a beneficial reduction in perceived stress (PSS) within the intervention site communities. Contrary to expectations, the interventions were associated with relative increases in PSS compared with the control group. However, differences in the results were found between those who did and did not visit nearby natural environments, including the intervention woodlands and other local green space, suggesting that stress levels within the communities reflected influences external to the intervention programme. In these high deprivation communities, other factors not captured by our study are likely to be stronger drivers of mental health outcomes and the interventions were insufficient to negate these likely other influences.

In an attempt to identify community-level factors not included in the study design that may have influenced these outcomes, the researchers re-contacted local authority officials and reviewed local newspaper reports for the period between 2012 and 2014. There was evidence of major urban redevelopment in one of the intervention sites during the course of our study, not notified to us by the local authority at the start of the study, including new community, shopping and leisure facilities, as well as housing redevelopment (including social housing) that required many people to move house within the community. No other potentially relevant external factors were identified at the community level.

Our data allow for the possibility that visits to natural environments may somewhat mitigate the effects of stress and stress-inducing factors, since the increase in stress over time in the intervention group was considerably greater for individuals who had *not* made nature visits (i.e., to woodlands and other nearby green spaces) in the previous year, compared with those who had made nature visits.

We did not find any significant gender differences in intervention effects. However, we did find an effect of distance from local woodlands, with differences in stress between intervention and control groups largest among those living furthest away from the woodland sites. There were significantly higher stress levels in the intervention group for those living further than 500 m from their local woodlands. This points to a potential threshold effect of approximately 500 m, which supports findings from many other studies on the beneficial effect of nearby access to nature [1].

There was some evidence of the interventions being associated with other health and wellbeing outcomes. Measures of PA showed a positive and significant association with the intervention group for moderate intensity activity, compared with the control group, where levels of PA decreased over time. This supports previous research reviewing the benefits of WIAT based on a meta-analysis of data from several different community surveys, which found evidence of an increase in reported visits to woodlands post-intervention and therefore in time spent walking associated with woodland visits [44]. However, in our study this effect, as with the increase in stress levels, appears to be due to external factors, since activities in natural environments in the intervention sample were not associated with this effect. A new leisure centre opened in one intervention site at the start of the study which may partly explain results.

There was evidence of an increase in connectedness to nature by Wave 3 of the survey for the intervention group compared to the control, and a similar comparative increase in social cohesion in the intervention group, significant both in Wave 2 and Wave 3. These findings were not significant in the cohort sample, suggesting either that they are not robust or that the cohort was not sufficiently powered to detect the observed changes. It is possible they reflect a small progression along pathways theorized to contribute to mental wellbeing, since nature connectedness has been correlated with emotional and psychological well-being [21] and community cohesion to mental health [45].

Other subjective data collected from the local community assist in considering the influence of WIAT on the findings reported here. The environmental audits showed that the local woods post-intervention was rated significantly higher quality than at baseline and they were also rated higher quality than control community woods. Again, this reflects earlier findings on WIAT projects as enhancing perceptions of woodland quality [44]. However, while many of those volunteering to participate in our study’s focus groups enjoyed visiting the woods and had engaged in intervention activities, this did not reflect the experience of the wider community as revealed in the survey results; the survey data suggest the social interventions only attracted [44] c. 2% of the local population. Furthermore, the focus group responses showed that short-term gains in environmental quality achieved through the WIAT interventions were often not sustained at the same level after the study finished in 2015, when Forestry Commission Scotland (FCS) were no longer responsible for management. Reflections on this by the FCS staff involved in WIAT and the study interventions suggest that a longer period of social engagement is needed—several years rather than the nine months of this study—before any significant change in woodland use is achieved at the community level [46]. There is also clearly a need for long term maintenance of the intervention sites by the landowner, and effective handover of such responsibilities, once any WIAT project is finished. While this did not appear to have happened effectively within the timeframe of the study, it is notable that, three years after project completion, the local authority owner of one intervention woodland supported its transfer to a community development trust so that the local community can manage the land for their collective benefit. This was accompanied by a grant to invest in further improvements and designed to ensure long term management commitments. Such action reflects recommendations from an earlier study of WIAT which proposed maintaining community involvement through ‘pulsed’ interventions over time, designed to refresh interest in local woodlands, and advocated removing barriers to community groups becoming involved with the financial management of woodlands [44].

In terms of economic evaluation, although there was no significant intervention effect for health-related quality of life, the cost-consequences analysis shows that the WIAT interventions are of low cost, based on the average per person, and have the potential to provide health and wellbeing benefits (e.g., for the secondary health outcomes identified) that are relatively cost-effective. The exploratory cost-per-QALY analysis showed that a wide range of cost-per-QALY figures would be consistent with the WIAT interventions. At an average cost per subject of just £12 for the interventions, and assuming a societal willingness to pay of at least £10,000 per QALY, then the interventions would only have to generate lifetime QALYs of 0.0012 on average for the interventions to be cost-effective—and this captures only the health benefits of the interventions. It is quite possible that the amenity value and other ecosystem services (e.g., contributions to biodiversity and sustainable urban drainage) of the woodlands alone could justify the investment.

The findings suggest that neither small-scale physical interventions alone, nor the addition of short-term community engagement interventions, are sufficient to make a measurable difference to community mental health and quality of life when applied to natural environments associated with deprived urban neighbourhoods. There are a number of avenues that might be explored further to help explain this finding, and consider what level of intervention (physical or social) is sufficient to affect community-level change in mental health outcomes. The costs of the interventions under WIAT were very modest and it is possible that a long term commitment to physical maintenance and community engagement may be needed to achieve significant health benefits, as other analyses of WIAT have suggested (ref). Health is both the product of, and part of, a complex system, with influences that operate dynamically across the life-course. Short-term interventions such as WIAT may be unable to affect health outcomes driven by wider, life-long factors and this issue may be particularly pertinent in deprived communities [47]. For example, there is evidence that attitudes to, and use of, natural environments in adulthood are strongly predicted by the frequency of access to such environments in childhood [48] and that childhood access to public green space may interact with socio-economic status to show effects for mental health that last a lifetime [49]. Such issues challenge linear models of environment-health causality [50]. They also suggest approaching community engagement via activities for children may produce more effective changes in woodland visiting behaviour (which can also influence parents and grandparents), rather than attempting to target adults directly to change established patterns of behaviour in later life.

## 8 Limitations

Although our study design drew on the existing relevant literature, both in terms of calculations of sample size and in choice of outcome measures, the lack of directly comparable studies based on quasi-experimental designs or natural experiments means that there may be limitations in terms of study power and sensitivity of measures used.

There were a number of challenges relating to the quasi-experimental design of this study that are common to many evaluations of environmental interventions. We found differences in our baseline sample population that may have influenced the outcome, such as poorer educational attainment and ability to cope on current income, and greater distance to local woods, among our intervention group compared to the control. We attempted to adjust for this but were restricted by collected survey data. It is possible that the results reflect interventions and external influences for which we have no data.

Our study was also restricted to three intervention and three control sites. It is therefore difficult to account for any variability in the success of the interventions between sites and to know whether the ‘typical’ effect of such interventions has been seen. The timeframe for implementation of the interventions and for the community surveys was also limited by the experimental design and it is possible that different PSS or other health effects might have been found after a longer period post-intervention.

Study recruitment is extremely challenging in deprived communities [51]. Although the overall response rate of 53% was lower than targeted, cooperation rates at each wave of the survey were at least 63% with a total cooperation rate of 70%. However, the recruitment of a longitudinal cohort within the sample was, as predicted, considerably lower (response rate 17%) than the cross-sectional survey recruitment.

## 9 Conclusions

Our study is the first controlled prospective study, where planned interventions to enhance urban populations’ access to natural environments provided the opportunity for a quasi-experimental design to evaluate the health and quality of life impacts.

Our evaluation involved primary data collection, a nested longitudinal component, and a mixed-method approach. A single evaluation such as this may not be definitive in all aspects but, as the first of its kind in relation to natural environments near disadvantaged urban communities, it demonstrates the feasibility of such research and contributes to a bigger picture of the impact of environmental interventions on health.

The findings show that the interventions were associated with comparative increases in stress, and moderate PA, but not in health-related quality of life. They were also associated with increased visits to nearby natural environments, and these nature visits appeared to moderate the stress outcomes. We found no evidence of an association between PA and nature visits. The results show that the reach of physical environmental interventions under WIAT is small, despite the associated WIAT programme of community engagement activities. The costs of the interventions were modest and it is possible that greater commitment to funding community engagement, and a longer time frame, is needed before significant health benefits can be achieved. Our study suggests that planning such interventions in future must also take into account land stewardship and the options available for ensuring long-term commitment to the management of forests and woodlands for health and wellbeing benefits. The example of community asset transfer now available under Scottish legislation might offer one model for future consideration, although its effectiveness has yet to be assessed.

There is global interest in finding affordable ways to enhance the environment to achieve population health benefits [1]. While evaluating the effects of environmental interventions is challenging, our study offers insights into how natural environment interventions might effectively be evaluated over time and in turn inform government policy-makers, land owners, stewardship communities, practitioners and non-government organisations engaged in supporting community health and wellbeing. We recommend that future studies of this sort consider a larger number of sites than six but, given the high cost of primary data collection, we also recommend the use of routinely collected data wherever possible, rather than relying on primary data collection. Further, we suggest that environmental interventions evaluated for health-related outcomes are considered over a longer period than two years, since this seems likely to reflect timescales relevant for observing changes in health at a community level.

Ultimately, our study relates to broader issues of the ways in which health and wellbeing issues are implicitly or explicitly addressed in planning practice. In a comparative study of England and Germany, Heiland et al. (2019) have shown that the potentials for including health issues in landscape planning are rarely used in practice, despite their mention in guidance and enabling legislation [4]. One example of a recent attempt to overcome disciplinary and professional silos is the 2015 development of the Place Standard Tool, jointly promoted by the Scottish Government, the National Health Service, Scotland, and Architecture and Design Scotland—the Government body responsible for promoting policy on architecture and ‘place’. However, the results of the implementation of such policy guidance remain to be seen.

## Supplementary Material

Appendix

## Figures and Tables

**Table 1:** Baseline characteristics of participants (imputed data).

Characteristic	Intervention N = 1061	Control N = 1056	Total N = 2117	

	%	%	%	p-Value for Test of Difference ^a^
**Age**				
16–24	9.1	7.9	9	0.33
25–34	18.4	14.5	16.4	**0.02**
35–44	16	14.3	15.2	0.27
45–54	19.4	20.1	19.7	0.67
55–64	12.1	16.7	14.4	**0.002**
65–74	18.5	20.2	19.3	0.32
75+	6.5	6.2	6.4	0.78
**Gender**				
Female	61.3	62.2	61.8	0.68
Male	38.6	37.8	38.2	0.68
**Life events**				
Better than normal	7.3	7.8	7.5	0.66
Much worse than normal	9.5	12.6	11.1	**0.02**
No different than normal	25	23.3	24.1	0.37
Nothing has happened in the last 12 months	58.2	56.3	57.3	0.38
**Social Class ^b^**				
Social Class I	2.5	4.1	3.3	**0.04**
Social Class II	18.7	21.9	19.2	**0.002**
Social Class III	18.7	19.6	19.1	0.59
Social Class IV	25	22.3	23.7	0.16
Social Class V	37.3	32.1	34.7	**0.01**
**Highest level of Qualification ^c^**				
No qualification	41.4	32.8	37.1	**<0.001**
Level 1	30.6	38	34.3	**<0.001**
Level 2	17.4	12.5	14.9	**0.002**
Level 3	7	9.2	8.1	0.06
Level 4	3.7	7.5	5.6	**<0.001**
**Working Status**				
No	56.1	58.5	57.3	0.26
Yes	43.4	41.5	42.7	0.26
**Income coping**				
Finding it difficult on present income	25	17.8	21.4	**<0.001**
Coping on present income	53.2	54.4	53.8	0.58
Living comfortably on present income	21.9	27.8	24.9	**0.002**
**Distance from woods**				
150m	6.5	26.2	16.3	**<0.001**
300m	12	26.4	19.2	**<0.001**
500m	15.7	24.7	20.2	**<0.001**
750m	31	15.2	23.1	**<0.001**
1500m	34.8	7.4	21.1	**<0.001**
**Access to a car**				
No	44.9	32.8	38.8	**<0.001**
Yes	55.1	67.2	61.2	**<0.001**
**Smoking status**				
Currently smoke	40.8	28.2	34.5	**<0.001**
Smoked in the past	21.1	18	19.6	0.08
Never smoked	38.1	53.8	45.9	**<0.001**
**Disability**				
No	86.6	88.6	87.6	0.16
Yes	13.4	11.4	12.4	0.16
**Health limited**				
Yes, limited a lot	8.8	11.3	10	0.06
Yes, limited a little	19.7	16.7	18.2	0.07
No, not limited at all	71.5	72.1	71.8	0.79
**Dog ownership**				
No	77.5	73	75.2	**0.02**
Yes	22.5	27	24.8	**0.02**
**Children in household**				
No	70.8	70.9	70.9	0.96
Yes	29.1	29	29.1	0.96
**Site Pair**				
Pair A	33.7	33.1	33.4	0.77
Pair B	33.2	33.1	33.2	0.98
Pair C	33.1	33.7	33.4	0.76

Notes:

a *p* values for test of differences <0.05 indicated in bold.

b Based on occupational categories, where I = Highest grade occupations; V = State pensioners, unemployed or lowest grade occupations.

c Level 4 represents Higher Education (first degree or higher).

**Table 2:** Intervention effects on the primary outcome (stress) and secondary outcomes: Health-related quality of life, physical activity, awareness of woodlands, woodland visit frequency and length, visits to nature, connectedness to nature and social cohesion (*n* = 5460).

Adjusted Models	Wave 2	Wave 3	Wald Test

Outcomes	β or OR (95% CI)	β or OR (95% CI)	*p*-Value
(a) Stress (PSS)	OR 1.52 (0.78 to 2.27)	3.58 (2.85 to 4.31)	<0.001
(b) EQ-5D ^a^	0.017 (−0.007 to 0.040)	−0.007 (−0.030 to 0.016)	0.14
(c.i) Vigorous PA ^b^	−152.9 (−422.6 to 116.8)	221.2 (−43.46 to 485.9)	0.03
(c.ii) Moderate PA ^b^	−215.4 ** (−409.4 to −21.39)	249.2 ** (58.25 to 440.1)	<0.001
(c.iii) Walking activity ^b^	203.3 ** (36.81 to 369.8)	−40.87 (−204.5 to 122.8)	0.01
(c.iv) Overall PA ^b^	−282.4 (−732.1 to 167.3)	275.2 (−163.2 to 713.5)	0.07
(d.i) Awareness of local woods	OR 2.26 (1.58 to 3.22)	OR 3.1 (2.15 to 4.46)	<0.001
(d.ii) Frequency of woodland visits (summer) ^c^	OR 0.79 (0.43 to 1.45)	OR 1.07 (0.57 to 1.99)	0.63
(d.iii) Frequency of woodland visits (winter) ^c^	OR 1.45 (0.66 to 3.18)	OR 0.82 (0.38 to 1.77)	0.42
(d.iv) Length of woodland visits ^c^	OR 0.43 (0.18 to 1.02)	OR 0.83 (0.36 to 1.90)	0.15
(d.v) Nature visits ^d^	OR 1.33 (0.94 to 1.88)	OR 2.69 *** (1.9 to 3.81)	<0.001
I Connectedness to nature	−0.19 * (−0.38 to −0.01)	0.39 *** (0.2 to 0.57)	<0.001
(f) Social cohesion	0.44 *** (0.22 to 0.65)	0.5 *** (0.29 to 0.7)	<0.001

**Notes**: Each row reports interaction coefficients of Type of Site and Wave for separate adjusted models. Measures for items(a) and (d.i) to (d.v) were set in binary form, with logistic regressions used and reported in terms of *Odds Ratio (OR)*.

a Health-related quality of life (EQ-5D).

b Physical activity (PA) model estimates shown in terms of Metabolic Equivalent (MET)-min/week.

c models using a reduced sample size, since only participants who had visited the target woodland areas for the study were included (*n* = 1393).

d a combined measure of visits to the target local woods or to other local green space or woods. * *p* < 0.5, ** *p* < 0.01, *** *p* < 0.0010. *p* values for test of differences <0.05 indicated in bold.

**Table 3:** Costing of the WIAT interventions.

Intervention Site	Description of Cost	Physical Intervention	Social Intervention	Total
**A**	Internal FCS time	£12,060	£3,922	
	External costs	£20,652	£16,126	
**Subtotal**		**£32,712**	**£20,048**	**£52,760**
**B**	Internal FCS time	£15,150	£32,024	
	External costs	£49,087	£16,066	
**Subtotal**		**£64,237**	**£48,090**	**£112,327**
**C**	Internal FCS time	£14,936	£12,052	
	External costs	£45,374	£4,218	
**Subtotal**		**£60,310**	**£16,270**	**£76,580**
**Total Cost**		**£157,259**	**£84,408**	**£241,667**

*p* values for test of differences <0.05 indicated in bold.
