# Supplementary material for: Enhancing Health Through Access to Nature: How Effective are Interventions in Woodlands in Deprived Urban Communities? A Quasi-experimental Study in Scotland, UK
Source: Sustainability. Author manuscript; Available in PMC 2019 Dec 16. (PMC6914372; doi:10.3390/su11123317)
Supplement: Appendix [file EMS85127-supplement-Appendix.pdf]

Appendix A. Notes on the EuroQol Measure (EQ-5D) Used in the Study

On the EQ-5D instrument for measuring health-related quality of life, two different versions were used: the original 3L version in Wave 1 and the new 5L version in Wave 2 and 3. This was potentially problematic because these instruments result in different health profile indices, hence their utilities might not be directly comparable. To ensure that the utilities from the two versions were consistent in all the three waves, and in the absence of the utility value sets for the 5L version for the wider UK population at the time of analysis, a cross-walk mapping approach was undertaken [30]. While the EQ-5D results were consistent with overall results, recently, it has been found that improvements in health-related quality of life are valued less with the 5L than the 3L version with implications on economic evaluation [52].

For full details of the economic evaluation methods and calculations, see the full study report [18].

Appendix B. Sensitivity Analyses Using the Cohort Sample (*n* = 609)

As a test of sensitivity, the longitudinal cohort sample was analysed using the same approach as for the full, cross-sectional sample. The primary outcome results using this cohort showed a similar comparative increase in stress (PSS) in the intervention group, although this was only significant at Wave 3 (PSS 3.03, 95% CI 1.54 to 4.52). Differences in PSS by gender were not significant for the cohort, similar to the full sample. Differences in PSS by distance from woods were found to be greatest, as for the full sample, within the upper distance bands (500–750 m and 750–1500 m) and were significant for these distance bands at Wave 3, with a difference PSS score of 4.45 for 500–750 m distance (95% CI 1.66 to 7.24) and 6.31 for 750–1500 m distance (95% CI 2.18 to 10.44).

As with the full sample, the cohort analysis showed a significant intervention effect in relation to increased awareness of local (target) woods (OR 3.39 (95% CI 1.72 to 6.67) and increased nature visits (OR 2.77, 95% CI 1.45 to 5.29) by Wave 3. The cohort analysis also confirmed the findings on PSS difference by nature visits, showing no significant PSS difference at Wave 2 or Wave 3 among the intervention group who visited nature, but a significant increase in PSS at both waves among intervention site participants who did not visit natural environments.

The cohort analysis showed a significant increase in moderate levels of physical activity and in total physical activity by Wave 3 in the intervention compared to control group but, again, we did not find significant changes in PA conditioned on nature visits. There were no significant intervention effects found for connectedness to nature or social cohesion in the cohort, unlike the full sample.

For the economic analysis, the sensitivity analysis results using the cohort sample showed that the majority of the simulated incremental costs and QALYs were positive in Wave 2 for the physical interventions and in Wave 3 for both physical and social interventions. This resulted in positive ICERs in both Waves.
